# A nomogram to predict the high-risk RS in HR+/HER2-breast cancer patients older than 50 years of age

**DOI:** 10.1186/s12967-021-02743-3

**Published:** 2021-02-16

**Authors:** Jing Yu, Jiayi Wu, Ou Huang, Jianrong He, Li Zhu, Weiguo Chen, Yafen Li, Xiaosong Chen, Kunwei Shen

**Affiliations:** Department of General Surgery, Comprehensive Breast Health Center, Ruijin Hospital, Shanghai Jiao Tong University School of Medicine, 197 Ruijin Er Road, Shanghai, 200025 China

**Keywords:** Breast cancer, The 21-gene recurrence score, Nomogram, Predict

## Abstract

**Background:**

The 21-gene recurrence score (RS) testing can predict the prognosis for luminal breast cancer patients. Meanwhile, patients > 50 years with RS > 25 have improved survival with adjuvant chemotherapy. The current study aimed to develop a nomogram with routine parameters to predict RS.

**Methods:**

We included patients diagnosed with hormone receptor (HR)-positive, human epidermal growth factor receptor-2 (HER2)-negative who underwent the 21-gene RS testing and aged > 50 years. The primary outcome was high-risk RS (> 25). Univariate and multivariate analyses were performed to identify significant predictors. A predictive nomogram based on logistic model was developed and evaluated with receiver operating characteristic (ROC) curves. The nomogram was internally validated for discrimination and calibration with bootstrapping method, and externally validated in another cohort. We then assessed the nomogram in different subgroups of patients and compared it with several published models.

**Results:**

A total of 1100 patients were included. Five clinicopathological parameters were used as predictors of a high-risk RS, including tumor grade, histologic subtype, ER expression, PR expression, and Ki-67 index. The area under the curve (AUC) was 0.798 (95% CI 0.772–0.825) and optimism adjusted AUC was 0.794 (95% CI 0.781–0.822). External validation demonstrated an AUC value of 0.746 (95% CI 0.685–0.807), which had no significant difference with the training cohort (P = 0.124). Calibration plots indicated that the nomogram-predicted results were well fitted to the actual outcomes in both internal and external validation. The nomogram had better discriminate ability in patients who had tumors > 2 cm (AUC = 0.847, 95% CI 0.804–0.890). When compared with four other existing models, similar AUC was observed between our nomogram and the model constructed by discriminate Lee et al.

**Conclusions:**

We developed a user-friendly nomogram to predict the high-risk RS in luminal breast cancer patients who were older than 50 years of age, which could guide treatment decision making for those who have no access to the 21-gene RS testing.

## Background

Breast cancer is the most frequent malignant disease in women. Treatment options for breast cancer used to depend on routine clinical and pathological characteristics of patients. However, the biological heterogeneity of the breast tumor could lead to a different response to treatment and prognosis in patients with similar clinicopathological features [1]. In the past few generations, the advent of microarray-based gene expression profiling contributed greatly to decipher the tumor heterogeneity, and several multigene signatures had been validated for risk assessment and efficacy prediction [2, 3].

The 21-gene recurrence score (RS) testing is a multigene assay performed on formalin-fixed paraffin-embedded sections by using a quantitative reverse transcriptase-polymerase chain reaction (RT-PCR) method [4]. The prognostic value of RS had been validated in hormone receptor (HR)-positive, human epidermal growth factor receptor-2 (HER2)-negative, 0–3 lymph node-involved breast cancer patients [4]. It could also predict the treatment effect of additional chemotherapy in patients who have received endocrine therapy [5]. Noticeably, the results of large prospective trial TAILORx confirmed that patients older than 50 years of age could spare the cytotoxicity of chemotherapy if they have RS ≤ 25 [6].

The 21-gene RS could provide vital information in the treatment decision-making process. Whereas this multigene assay was not available in every part of the world, and not all patients could afford the expensive test. Those lead to that only a small part of eligible patients received this genomic testing in the real world [7]. So, there’s an urgent need for alternatives that have the potential to provide similar predictive information as RS in resource-constrained settings, in order to guide treatment selection.

Up to now, there were several models published to predict RS risk, while most of them neglected that patients ≤ 50 years could still to some extent benefit from chemotherapy with RS ≤ 25 [6]. In the current study, we aim to construct a nomogram with routine clinicopathological parameters to predict the high-risk RS, and we limited the application of such a nomogram to patients ≥ 50 years, for whom the benefit of chemotherapy is sound and solid. We also compared our nomogram with other existing models. Via this nomogram, we hope to help the patients who have no access to the 21-gene RS testing judge whether to receive adjuvant chemotherapy or not.

## Methods

### Patients selection and data processing

Patient diagnosed with primary breast cancer between January 2009 and February 2020 and received breast operation at Ruijin Hospital, Shanghai were retrospectively enrolled into training and internal validation cohort. Data on clinicopathological characteristics and 21-gene RS were retrieved from Shanghai Jiao Tong University Breast Cancer Database (SJTU-BCDB). Patients were included if they met the following criteria: (1) HR + /HER2- primary invasive breast cancer; (2) underwent the 21-gene testing; Exclusion criteria were as follow: (1) lymph-node positive; (2) male patients; (3) had HER2 + tumors, triple-negative breast cancer, or ductal carcinoma in situ; (4) had tumors with favorable histological subtype (including mucinous carcinoma, solid papillary carcinoma, papillary carcinoma, encapsulated papillary carcinoma, neuroendocrine carcinoma, tubular carcinoma); (5) had missing data of clinicopathological parameters; (6) patients who were ≤ 50 years of age.

External validation cohort consisted of 77 patients diagnosed at other breast centers and referred to receiving 21-gene RS testing, and 205 patients diagnosed at Ruijin hospital after February 2020.

### Pathological and immunochemistry (IHC) analysis

Pathological and IHC analysis was accomplished at the Department of Pathology of Ruijin Hospital by experienced pathologists. Examination of histological subtype, tumor grade, and lymph vascular invasion (LVI) was referring to the World Health Organization classification [8]. Expression of estrogen receptor (ER), progesterone receptor (PR) and Ki-67 index were evaluated by IHC analysis on 4-μm-thick, formalin-fixed, paraffin-embedded (FFPE) tissue sections with the following antibodies: ER (clone 1D5; 1:100; rabbit monoclonal; Dako; Agilent Technologies, Inc.), PR (clone PR636; 1:100; mouse monoclonal; Dako; Agilent Technologies, Inc.) and Ki-67 (clone MIB-1; 1:100; mouse monoclonal; Dako; Agilent Technologies, Inc.). Luminal subtype was identified according to the 2013 St.Gallen expert consensus [9].

### The 21-gene RS testing

The 21-gene RS testing was performed on formalin-fixed, paraffin-embedded tissue sections as we previously described. In brief, hematoxylin and eosin-stained slides were used to identify enough tumor tissues. Then, total RNA was extracted by the RNeasy FFPE RNA kit (Qiagen GmbH) from two 10-μm unstained sections after verifying the absence of DNA contamination. Gene-specific reverse transcription was performed using the Omniscript RT kit (Qiagen GmbH) and standardized quantitative RT-PCR was accomplished in 96-well plates using an Applied Biosystems 7500 Real-Time PCR system (Thermo Fisher Scientific, Inc.). The expression levels of each gene were measured in triplicate, and expression levels of cancer-associated genes were normalized by 5 reference genes. Finally, the RS was calculated with a specific algorithm as previously described and categorized as low-risk (RS ≤ 25) and high-risk (> 25).

### Development and validation of the nomogram

The nomogram was developed according to the method published by Lasonos et al. [10]. Univariable and multivariable logistic analyses were performed for selecting the predictor variables, and the nomogram was established according to the coefficients of logit function. The receiver operating characteristic (ROC) curve with area under the curve (AUC) was used for measuring the discrimination capacity. The goodness of fit of the model was assessed by the Hosmer–Lemeshow test. The internal validation was performed by applying bootstrap resampling 1000 times, and optimism-corrected AUC was calculated by subtracting the optimism from the original AUC [11]. Calibration was assessed by calibration plot with 1000 bootstrap resampling. Subgroup analysis of the performance of the nomogram was conducted according to clinicopathological parameters. We further compared our nomogram with several published models predicting high-risk RS by using the ROC curve with AUC. The predictive performance of the nomogram was validated in an external cohort. The ROC curve and AUC were compared with the training cohort in order to assess the discriminative ability. Calibration was also evaluated.

## Statistical analysis

Distribution of the clinicopathological parameters according to RS risk stratification was evaluated by the chi-square test. Univariate and multivariate analysis to select predictor variables were performed with IBM SPSS Statistics (version 25.0). Comparisons of the AUC were assessed by Delong’s method. Two-sided P value < 0.05 was considered statistically significant. Statistical analyses and graphics for developing and validating the nomogram were accomplished with R software (version 3.6.3).

## Results

### Clinicopathological characteristics of patients

**Fig. 1:**
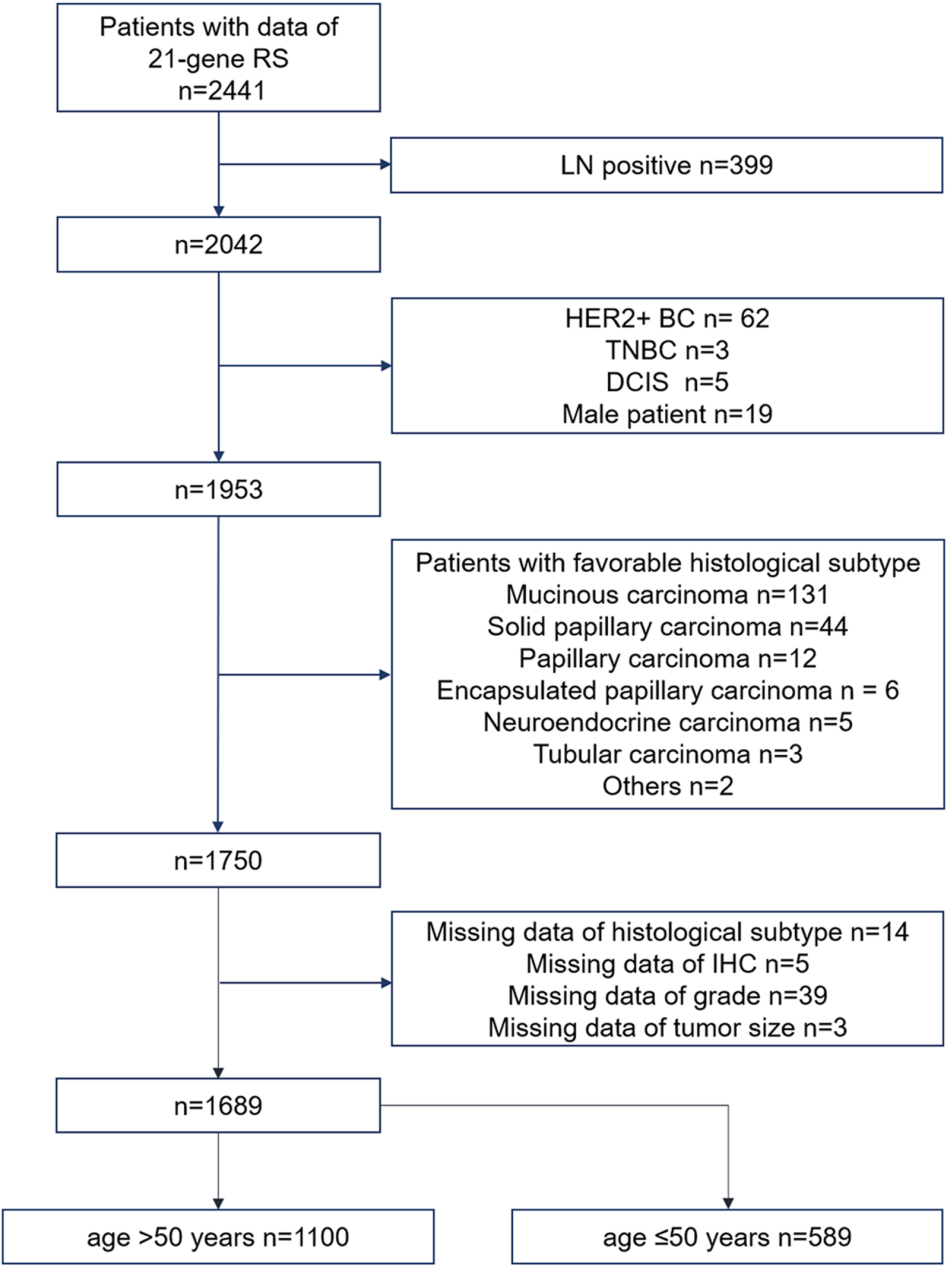
Flow chart

A total of 2441 patients with recurrence score results were reviewed and 1100 patients aged > 50 years were included eventually to develop the nomogram. The flow chart was shown in Fig. 1. Detailed baseline characteristics of the overall population were presented in Table 1. The mean age of overall patients was 63.33 ± 8.38, and 93% of the patients were menopausal. The mean tumor size was 1.93 ± 0.98, with 70.7% of tumors ≤ 2 cm. IDC was the most common histology (86.8%), other types accounted for 6.5%, and mixed tumors accounted for 6.6%. Mean values of ER expression, PR expression, and Ki-67 index were 89.24 ± 15.07, 47.08 ± 36.80, and 18.97 ± 16.16, respectively. Among all the patients, 69.8% had Luminal B-like tumors. The mean value of the recurrence score was 25.07 ± 10.21, and there were 511 patients (46.5%) had high-risk RS (> 25).

**Table 1:** baseline characteristics

	Total N (%)	RS low-risk N (%)	RS high-risk N (%)	P-value
Age				0.001
Mean ± SD	63.33 ± 8.38	64.13 ± 8.56	62.41 ± 8.09	
Menstrual status				0.193
Pre-	77 (7.0%)	47 (8.0%)	30 (5.9%)	
Post-	1023 (93.0%)	542 (92.0%)	481 (94.1%)	
Tumor size				0.017
≤ 2 cm	778 (70.7%)	435 (73.9%)	343 (67.1%)	
> 2 cm	322 (29.3%)	154 (26.1%)	168 (32.9%)	
Histologic subtype				0.006
IDC	955 (86.8%)	501 (85.1%)	454 (88.8%)	
Mixed	73 (6.6%)	52 (8.8%)	21 (4.1%)	
Others	72 (6.5%)	36 (6.1%)	36 (7.0%)	
Tumor grade				< 0.001
I	140 (12.7%)	102 (17.3%)	38 (7.4%)	
II	787 (71.5%)	428 (72.7%)	359 (70.3%)	
III	173 (15.7%)	59 (10.0%)	114 (22.3%)	
LVI				0.782
Yes	54 (4.9%)	30 (5.1%)	24 (4.7%)	
No	1046 (95.1%)	559 (94.9%)	487 (95.3%)	
ER status				< 0.001
Mean ± SD	89.24 ± 15.07	91.79 ± 11.08	86.30 ± 18.21	
PR status				< 0.001
Mean ± SD	47.08 ± 36.80	62.29 ± 33.68	29.55 ± 32.19	
Ki-67 index				< 0.001
Mean ± SD	18.97 ± 16.16	15.20 ± 12.73	23.32 ± 18.45	
Luminal subtype				< 0.001
Luminal-A like	332 (30.2%)	257 (43.6%)	75 (14.7%)	
Luminal-B like	768 (69.8%)	332 (56.4%)	436 (85.3%)	

### Nomogram development

Univariable analysis demonstrated that age (P = 0.001), tumor size (P = 0.017), histological subtype (P = 0.006), tumor grade (P < 0.001), ER expression (P < 0.001), PR expression (P < 0.001), Ki-67 index (P < 0.001), and Luminal subtype (P < 0.001) were associated with high-risk RS. In multivariable logistic regression analysis, histologic subtype (P = 0.015, Mixed types: OR = 0.41, 95% CI 0.19–0.90, P = 0.026; Other types: OR = 0.99, 95% CI 0.57–1.72, P = 0.974), tumor grade (P = 0.007; grade II: OR = 2.08, 95% CI 1.31–3.31, P = 0.002; grade III: OR = 2.29, 95% CI 1.24–4.25, P = 0.008), ER expression (OR = 0.99, 95% CI 0.98–0.999, P = 0.032), PR expression (OR = 0.97, 95% CI 0.97–0.98, P < 0.001), and Ki-67 index (OR = 1.03, 95% CI 1.02–1.04, P < 0.001) were still significantly associated with high-risk RS (Table 2). Ki-67 had the most significant impact on high-risk RS.

**Table 2:** Multivariate logistic regression analysis of variables

Variables	OR	95% CI	P value
Age (years)	0.98	0.97–1.00	0.066
Tumor size (> 2 cm vs. ≤ 2 cm)	1.12	0.82–1.52	0.487
Histological subtype			0.015
Mixed vs. IDC	0.41	0.19–0.90	0.026
Others vs. IDC	0.99	0.57–1.72	0.974
Tumor grade			0.007
Grade II vs. Grade I	2.08	1.31–3.31	0.002
Grade III vs. Grade I	2.29	1.24–4.25	0.008
ER expression	0.99	0.98–1.00	0.032
PR expression	0.97	0.97–0.98	< 0.001
KI67 index	1.03	1.02–1.04	< 0.001
Luminal subtype (Luminal A-like vs. Luminal B-like)	0.92	0.62–1.37	0.672

**Fig. 2:**
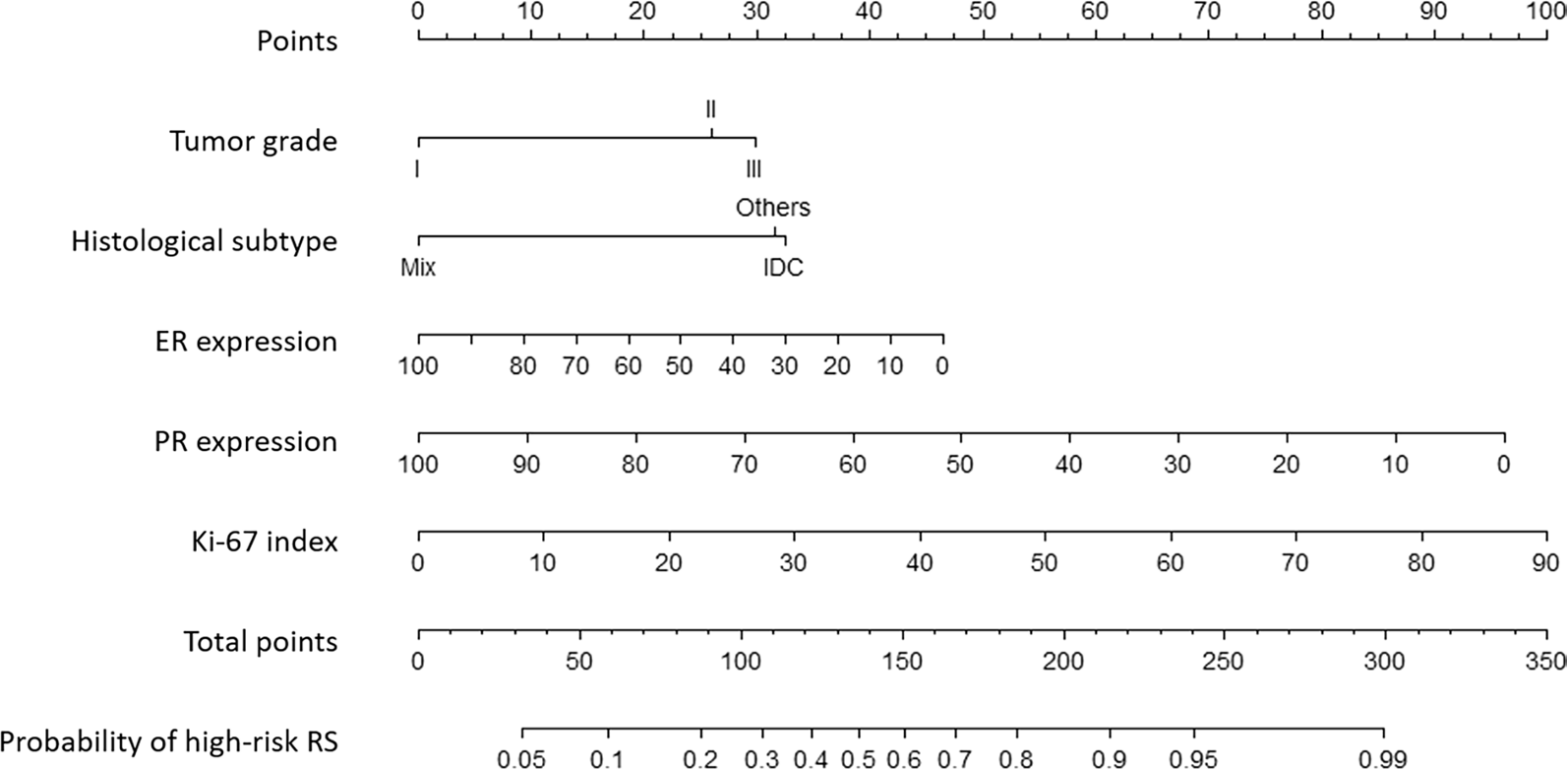
The nomogram predicting the probability of high-risk RS (RS > 25). A nomogram with tumor grade, histological subtype, age, ER expression, PR expression, and Ki-67 index predicting the probability of high-risk recurrence score (RS)

No significant collinearity was observed among the continuous variables. Variables that were statistically significant in the multivariable analysis were used to construct the nomogram. Regression coefficients for five variables and the intercept were presented in Additional file 1: Table S1. For each patient, the total point was calculated by adding up the score of each variable to predict the probability of having high-risk RS (Fig. 2).

### Performance of the nomogram and internal validation and external validation

**Fig. 3:**
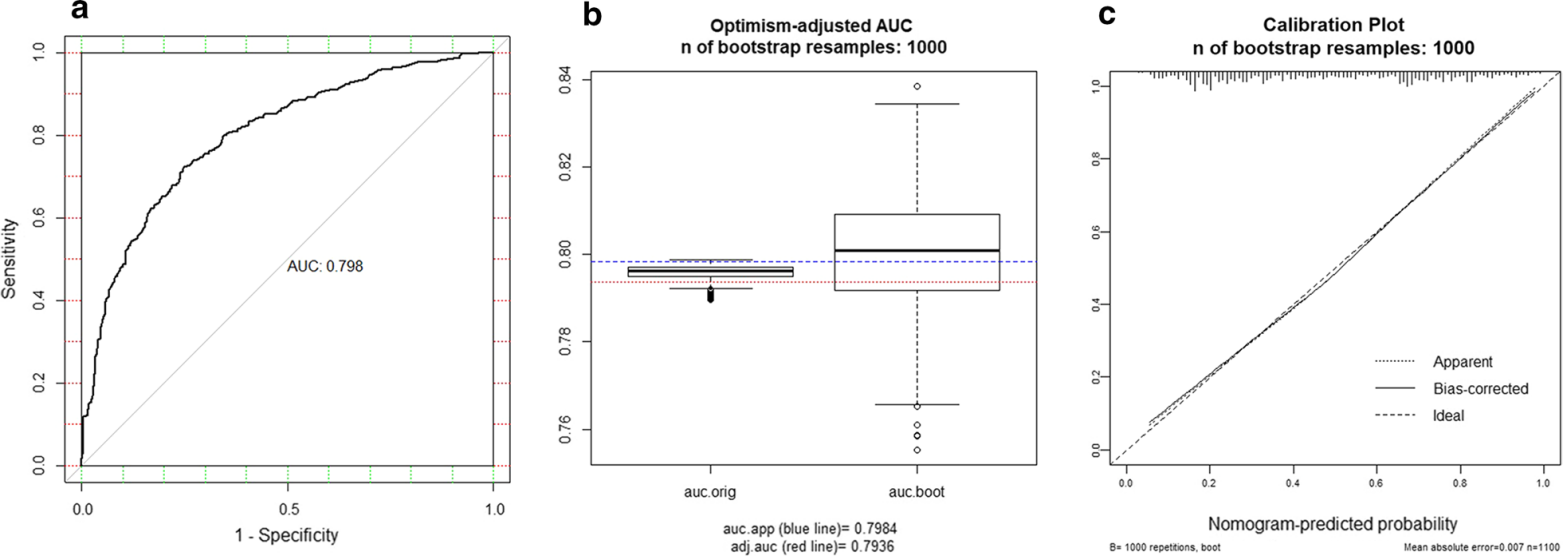
Discrimination of the nomogram and internal validation of the nomogram. **a** The discrimination assessed by ROC curve. The AUC is 0.798 (95% CI 0.772–0.825). **b** The nomogram was internally validated by applying bootstrap sampling for 1000 times. The optimism adjusted AUC is 0.794 (95% CI 0.781–0.822). **c** Calibration plot of the nomogram. The nomogram was calibrated for the probability of being high-risk RS. (bootstrap 1000 repetitions)

The AUC of the model was 0.798 with a 95% confidence interval (CI) ranging from 0.772 to 0.825, which indicated a strong model (Fig. 3a). The nomogram had good fitness with a P-value of 0.395 for the Hosmer–Lemeshow test. With the optimal cutoff value of 0.472, the overall accuracy was 73.7% with the sensitivity of 72.2%, the specificity of 75.0%, positive predictive value (PPV) of 71.5%, and negative predictive value (NPV) of 75.7% (Additional file 1: Table S2). Sensitivity, specificity, PPV, NPV according to different cutoff values were presented in Additional file 1: Table S3.

After bootstrap sampling for 1000 times, the optimism-adjusted AUC was 0.794 (95% CI 0.781–0.822, Fig. 3b). The calibration plot of the nomogram was shown in Fig. 3c, which illustrated that the predicted result had good consistency with the actual record.

A total of 282 patients were enrolled for external validation. The AUC was 0.746 (95% CI 0.685–0.807) in the external cohort, which had no significant difference with the training cohort (P = 0.124). The calibration plot was shown in Additional file 1: Figure S2 which indicated good calibration.

### Subgroup analysis of the discriminating ability of the nomogram

**Fig. 4:**
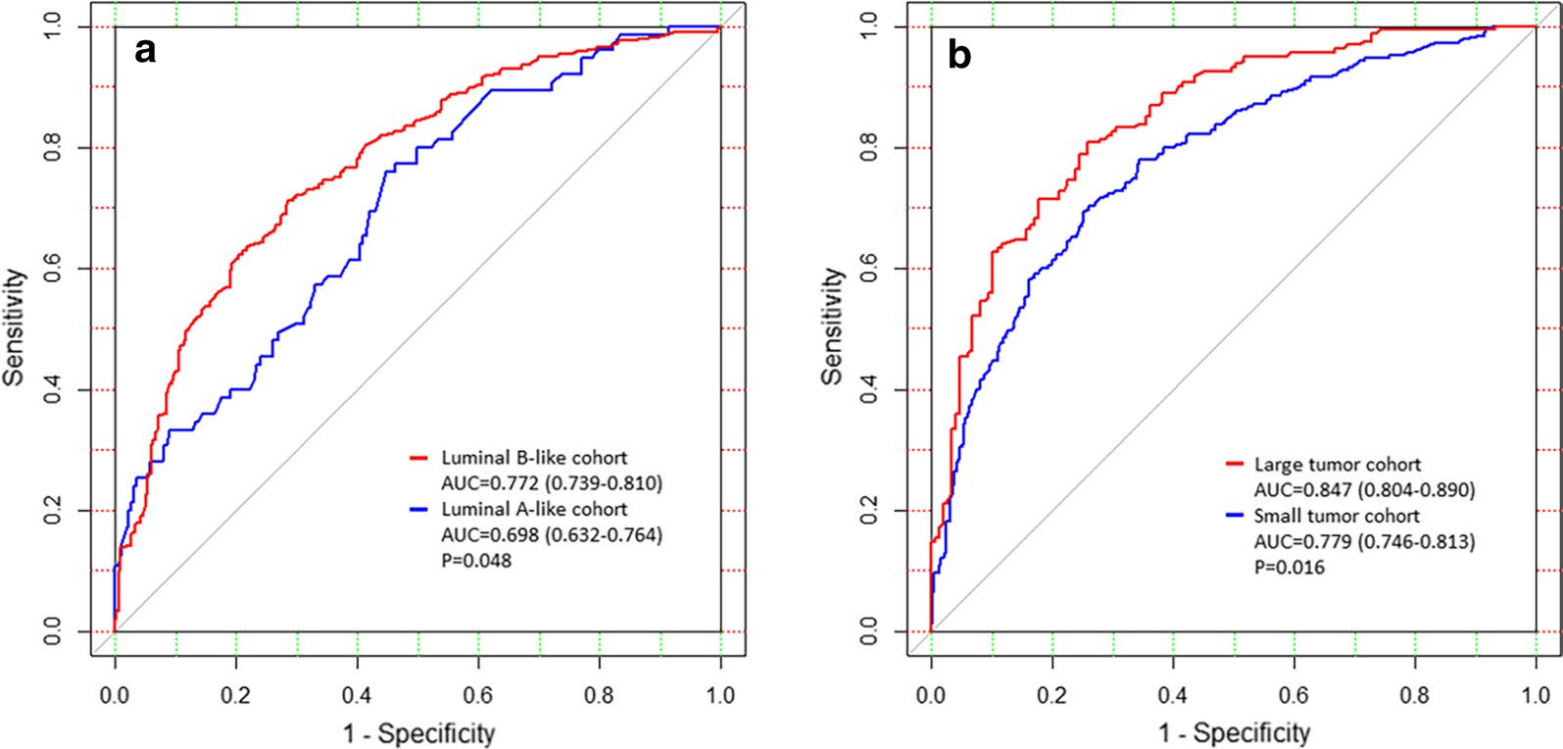
Subgroup analysis of discrimination of the nomogram. **a** Comparison of the nomogram in the Luminal B-like and the Luminal A-like cohort. Luminal B-like: The AUC is 0.772 (95% CI 0.739–0.810); Luminal A-like: The AUC is 0.698 (95% CI 0.632–0.764); Delong’s test P = 0.048. **b** Comparison of the nomogram in the large tumor cohort and the small tumor cohort. Large tumor: The AUC is 0.847 (95% CI 0.804–0.890); Small tumor: The AUC is 0.779 (95% CI 0.746–0.813); Delong’s test P = 0.016

We further applied the nomogram in different subgroups of patients to see if the nomogram has similar performance in population with different clinicopathological characteristics. When validating the nomogram classified by the luminal subtype, the AUC was 0.772 (95% CI 0.739–0.810) for the luminal B-like cohort and 0.698 (95% CI 0.632–0.764) for the Luminal A-like cohort (P = 0.048, Fig. 4a). And when using 2 cm as a cutoff to distinguish the large tumor with the small tumor, the AUC was 0.847 (95% CI 0.804–0.890) and 0.779 (95% CI 0.746–0.813) respectively (P = 0.016, Fig. 4b). We also evaluated our nomogram in patients ≤ 50 years of age. The AUC was 0.739 (95% CI 0.698–0.781), which was statistically significantly different from the older women cohort (P = 0.019, Additional file 1: Figure S1).

### Comparison of the nomogram with other existing published models

**Fig. 5:**
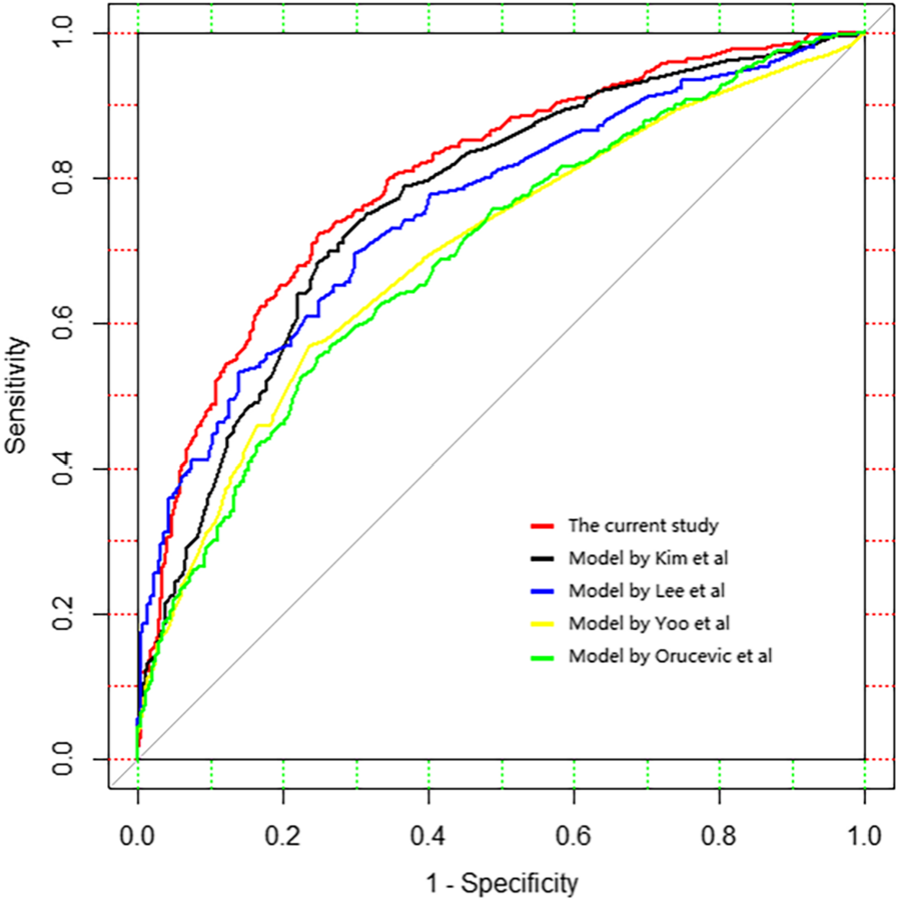
validation of the model predicting high-risk RS (RS > 25) with clinicopathological characteristics using the current database. The red curve is the ROC curve for the current study, the AUC is 0.798 (95% CI 0.772–0.825); The black curve represents the validation of the model constructed by Hyun-seok Kim et al. using our database, the AUC is 0.766 (95% CI 0.738–0.794), P < 0.001 (delong test); The blue curve represents the validation of the model constructed by Sae Byul Lee et al. using our database, the AUC is 0.757 (95% CI 0.717–0.797), P = 0.090 (delong test); The yellow curve represents the validation of the model constructed by Amila Orucevic et al. using our database, the AUC is 0.695 (95% CI 0.664–0.727), P < 0.001 (delong test); The green curve represents the validation of the model constructed by Shin Hye Yoo et al. using our database, the AUC is 0.699 (95% CI 0.668–0.730), P < 0.001 (delong test)

Discrimination ability of the current nomogram was then compared to four published models, which were also set up for predicting categorical RS risk. We selected those models because they use the TAILORx cutoff as the outcome as well. The summarization of models was listed in Table 3. It worth noting that those models used different predictors or different rules to score the predictors, and we reordered the data accordingly. Figure 5 illustrated the ROC curve for the four models and our nomogram. Only 554 out of 1100 patients who had results for ER and PR in terms of Allred score were used to validate the model constructed by Lee et al. and the AUC was 0.757 (95% CI 0.717–0.797) with no statistic difference from our nomogram (P = 0.090). The AUC was 0.766 (95% CI 0.738–0.794) for Kim’s model, 0.699 (95% CI 0.668–0.730) for models by Yoo et al., and 0.695 (95% CI 0.664–0.727) for the model by Orucevic et al. The latter three models were significantly different with our nomogram (P < 0.001).

**Table 3:** Summary of models predicting high-risk RS (RS > 25) with clinicopathological characteristics

Year	Author	Patients (n)	Predictors	Type of variables	Calibration	Discrimination
2016	Hyun-seok Kim et al	Training n = 1113	ER	Numerical (percent)	52.50%	/
		Validation n = 472	PR	Numerical (percent)		
			Ki-67	Numerical (percent)		
			HER2	Categorical (negative/positive)		
			Elston grade	Categorical (Low/Intermediate/High)		
2019	Amila Orucevic et al	Training n = 65,754	Age	Numerical	86.80%	0.81
		Validation n = 18,585	Size	Numerical		
			Grade	Categorical (1/2/3)		
			PR	Categorical (negative/positive)		
			Histology	Categorical (IDC/ILC/IDC + ILC/IDC + others)		
2019	Sae Byul Lee et al	Training n = 340	ER Allred score	Numerical (0–8)	/	0.90
		Validation n = 145	PR Allred score	Numerical (0–8)		
			Nuclear grade	Categorical (1/2/3)		
			LVI	Categorical (negative/positive)		
			Ki-67	Numerical (percent)		
2019	Shin Hye Yoo et al	Training n = 192	Nuclear grade	Categorical (Low-Intermediate/High)	/	0.856
		Validation n = 264	PR	Categorical (negative/positive)		
			Ki-67	Numerical (percent)		
The current study		Training n = 1100 1000 bootstrap internal validation, external validation n = 282	Histologic subtype	Categorical (IDC/mixed/others)		
			Grade	Categorical (1/2/3)		0.798
			ER	Numerical (percent)		
			PR	Numerical (percent)		
			Ki-67	Numerical (percent)

## Discussion

In the current study, we constructed a user-friendly nomogram with five routine clinicopathological predictors, including histologic subtype, tumor grade, ER expression, PR expression, and Ki-67 index. Validation of the nomogram demonstrated optimal predictive ability in terms of discrimination and calibration. Subgroup analysis showed that the nomogram had better performance in patients who had tumors larger than 2 cm. When compared with other published models, consistency was observed, indicating the value of our nomogram for clinic practice.

The 21-gene RS testing could provide more precise prognostic and predictive information when compared with classical clinicopathological parameters. However, this multigene assay is not available in some countries, the high price also prevented a lot of patients from receiving the test. Literature reported that only a quarter to a third of the eligible patients in the United States had this assay performed [7, 12], and in developing countries, certain controversy remained regarding the applicability of this testing [13]. On another hand, cost-effectiveness analysis demonstrated that the 21-gene RS testing was only associated with lower cost in patients with clinical high-risk [14, 15]. Thus, a surrogate for the 21-gene RS testing is needed for those who have no access to this assay, as well as to relieve the heavy financial burden of patients for whom the testing is not cost-efficient [16].

The NSABP B-20 retrospectively validated that patients with RS > 30 could have better distant recurrence-free survival if they received chemotherapy [17]. In the current study, we set RS > 25 as the objective of prediction because this was used for defining high-risk RS in the prospective TAILORx trial. We postulate that the chemotherapy should be included in treatment for patients with an RS of 26–30 since in the TAILORx trial those patients were assigned to use chemotherapy and had better clinical results than expected outcomes with endocrine monotherapy [18]. Moreover, we only included patients > 50 years, because in patients of 50 years of age or younger some chemotherapy benefit could be found in those had an RS of 16–25 [6]. To the best of our knowledge, there was no published model take the age stratification into consideration. We further validated our nomogram in patients ≤ 50 years, and the AUC was 0.739, which had a significant difference with that in the older cohort. The inapplicability of the current nomogram in the younger population may due to the biological difference of tumors between young and old breast cancer patients [19].

For the development of the nomogram, we used five variables: tumor grade, histologic subtype, ER expression, PR expression, and Ki67-index. Ki-67 index was the most significant predictor of high-risk RS, which was consistent with Lee et al. reported [20]. Indeed, serving as a proliferation index, Ki-67 had been universally recognized and has been endorsed to discriminate Luminal A-like with Luminal B-like breast cancer [9, 21]. However, further efforts are imperative to improve the poor interlaboratory reproducibility and resolve the disagreement during cutoff selection for this biomarker [22]. The relationship between RS and tumor grade as well as PR status were always reported, and those two parameters had been constantly incorporated in the model predicting high-risk RS [23–26]. Tumor grade is associated with the biologic aggressiveness of tumors, which is also the only one factor that showed a significant effect on prognosis beyond RS in the TAILORx. PR negativity together with the semi-quantitative measurement of PR such as Allred scoring were both correlated to RS stratification [20, 27]. We used the percentage of the positively stained cell as a rule for scoring ER and PR as Kim et al. because quantitative estrogen and progesterone receptor was validated to be associated with the risk of relapse [25, 28].

Our nomogram had an AUC of 0.798, indicating a strong model with good discrimination. And subgroup analysis demonstrated that the model had better performance in patients with large tumors. Tumor size was significantly associated with RS in the univariant analysis, but missed the statistical significance when entering the model together with other variables, while it was verified as a predictor in the model constructed by Orucevic et al. [26]. When stratified by the luminal subtype, the AUC values of two cohorts were both lower than that in the overall population. A possible explanation was that the categorization of the luminal subtype depends on PR expression and Ki-67 index, two major predictors of high-risk RS. And when grouped patients with these two parameters, the predictive value may be narrowed accordingly.

Up to now, there were several models using clinical parameters to estimate the RS with TAILORx cutoffs [20, 25, 26, 29, 30]. Our nomogram has a similar discriminative ability with models developed by Lee et al. [20]. Kim et al. used forest random method to develop a model and allows for online implementation of the model [25]. We used four predictors identical to them but didn’t include the HER2 status into the establishment of the nomogram. Although the 21-gene RS testing is only applicable in HR + /HER2− patients, literature reported that using quantitative RT-PCR to discriminate HER2 status could further elucidate the benefit of chemotherapy. Hence, other measurements of HER2 status such as Fluorescence In Situ Hybridization may contribute to a better model in further research [31]. Orucevic et al. built a nomogram using a large cohort in National Cancer Data Base (NCDB) with a C-index of 0.81, while the AUC was only 0.695 when validated with our patients [26]. The racial disparity may be one reasonable explanation. Meanwhile, Ki-67 was not regularly recorded in the NCDB thus was not incorporated into their model, whereas our study demonstrated the importance of this biomarker in predicting high-risk RS. Recently, Zhang et al. developed a model by using the Ki-67 index, PR expression, tumor grade, and tumor size with a predictive accuracy of 86.5%, which also had prognostic value [30]. However, it’s hard to perform a direct comparison between our two models because of the decision tree method they used.

Our strength was that we confined the scope of application to patients > 50 years, for whom the efficacy of chemotherapy is undoubted. There were also several limitations. First, as a retrospective study, selection bias may make the results less convincing, although all patients who met the criterion of 21-gene RS testing consecutively received this multigene assay in our center. Second, the majority of enrolled patients were Asians, and the nomogram needed to be validated in patients of other races. Using public databases with detailed ER, PR, and Ki67 expression levels for external validation may be an attractive strategy in the future. Last but not least, though using multivariate logistic regression, we developed a strong model with good fitness, other methods such as decision tree model and random forest model may also work, which warrant further consideration.

## Conclusions

In the current study, we used five routine items on the pathological reports to develop a nomogram predicting high-risk RS. With robust discrimination and calibration, the nomogram could help to make treatment options when the 21-gene RS testing is not available or affordable.

## Supplementary Information

**Additional file 1: Table S1.**Coefficients of the variables and intercept in multivariate logistic regression model. **Table S2. **Predictive ability of the nomogram for correct categorization of high-risk RS compared to the observed high-risk ODX test results using the optimal threshold. **Table S3. **Sensitivity, specificity, positive predictive and negative predictive values according to different cutoff values. **Figure S1. **Discrimination of nomogram in patients ≤50 years. **Figure S2.** Discrimination and calibration ability of the nomogram in external validation cohort

## Data Availability

The datasets used and/or analysed during the current study are available from the corresponding author on reasonable request.
