# Supplementary material for: A nomogram to predict the high-risk RS in HR+/HER2-breast cancer patients older than 50 years of age
Source: J Transl Med. 2021 Feb 16;19:75. doi: 10.1186/s12967-021-02743-3 (PMC7885620; doi:10.1186/s12967-021-02743-3)

**Table S1** coefficients of the variables and intercept in multivariate logistic regression model

| Variables | βvalue |
| --- | --- |
| Histological subtype |  |
| Mixed | -0.91873 |
| Others | -0.02598 |
| Tumor grade |  |
| Grade II | 0.73497 |
| Grade III | 0.84450 |
| ER expression | -0.01315 |
| PR expression | -0.02720 |
| KI67 index | 0.03140 |
| Intercept | 1.09462 |

**Table S2** Predictive ability of the nomogram for correct categorization of high-risk RS compared to the observed high-risk ODX test results using the optimal threshold

|  | Observed high-risk RS | |
| --- | --- | --- |
| Predicted high-risk RS | High | Low |
| High | 369 | 147 |
| Low | 142 | 442 |
| Overall accuracy | 73.7% | |

**Table S3** Sensitivity, specificity, positive predictive and negative predictive values according to different cutoff values

| probability | Sensitivity (%) | Specificity (%) | PPV (%) | NPV (%) |
| --- | --- | --- | --- | --- |
| 0.25 | 89.0 | 44.7 | 58.3 | 82.4 |
| 0.30 | 85.1 | 54.8 | 62.1 | 81.0 |
| 0.35 | 81.4 | 62.0 | 65.0 | 79.3 |
| 0.40 | 76.9 | 67.2 | 67.1 | 77.0 |
| 0.45 | 73.8 | 72.7 | 70.1 | 76.2 |
| 0.50 | 68.9 | 76.2 | 71.5 | 73.8 |
| 0.55 | 64.2 | 81.0 | 74.5 | 72.3 |
| 0.60 | 61.1 | 83.9 | 76.7 | 71.3 |
| 0.65 | 55.0 | 86.4 | 77.8 | 68.9 |
| 0.70 | 42.3 | 93.2 | 84.4 | 65.0 |

**Figure S1** Discrimination of nomogram in patients ≤50 years
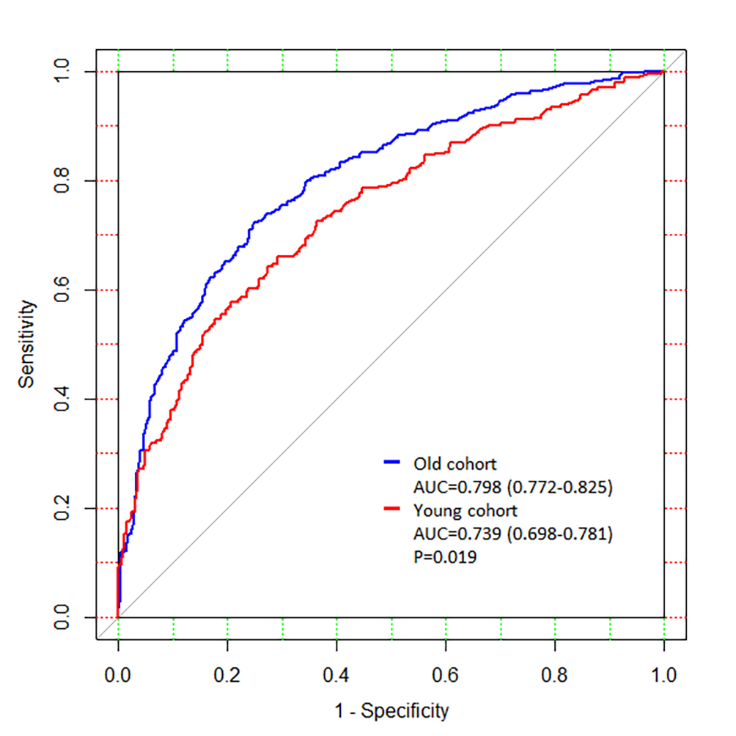


**Figure S2** Discrimination and calibration ability of the nomogram in external validation cohort


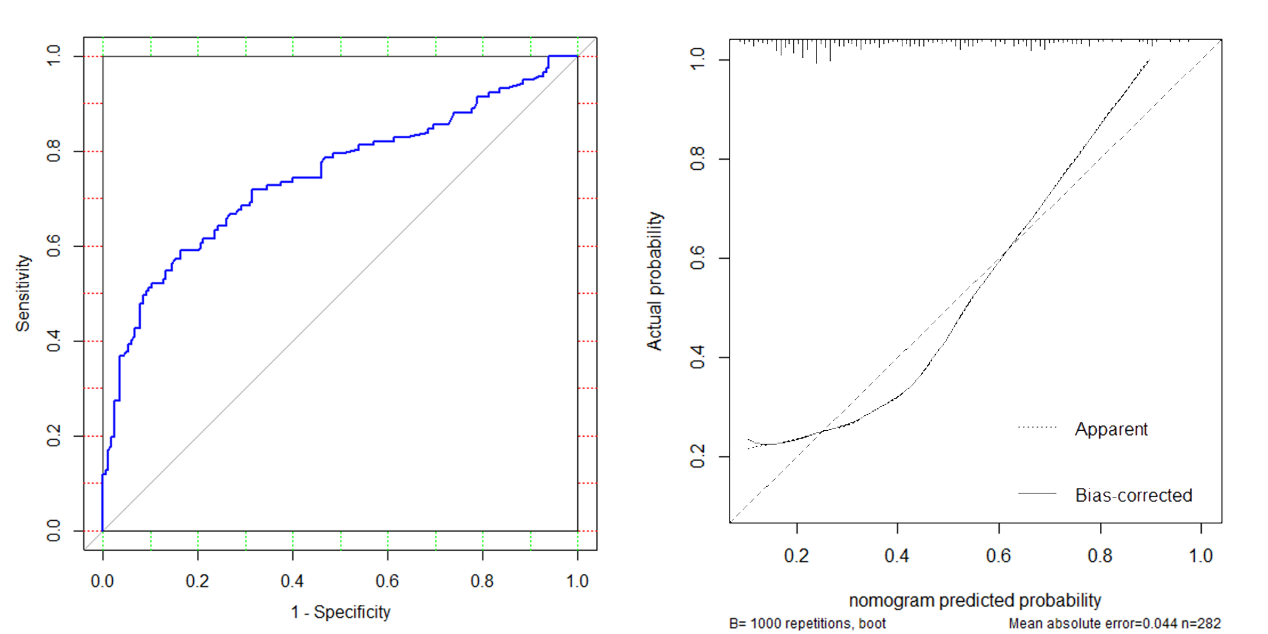

Supplement: Supplementary file 1 — Additional file 1: Table S1.Coefficients of the variables and intercept in multivariate logistic regression model. Table S2. Predictive ability of the nomogram for correct categorization of high-risk RS compared to the observed high-risk ODX test results using the optimal threshold. Table S3. Sensitivity, specificity, positive predictive and negative predictive values according to different cutoff values. Figure S1. Discrimination of nomogram in patients ≤50 years. Figure S2. Discrimination and calibration ability of the nomogram in external validation cohort [file 12967_2021_2743_MOESM1_ESM.docx]
